# CD5 molecule-like and transthyretin as putative biomarkers of chronic myeloid leukemia - an insight from the proteomic analysis of human plasma

**DOI:** 10.1038/srep40943

**Published:** 2017-01-24

**Authors:** Iram Fatima, Saima Sadaf, Syed Ghulam Musharraf, Naghma Hashmi, Muhammad Waheed Akhtar

**Affiliations:** 1 School of Biological Sciences, University of the Punjab, Lahore-54590, Pakistan; 2 Institute of Biochemistry and Biotechnology, University of the Punjab, Lahore-54590, Pakistan; 3 Dr Panjwani Center for Molecular Medicine and Drug Research, International Center for Chemical and Biological Sciences, University of Karachi, Karachi-75270, Pakistan

## Abstract

Better and sensitive biomarkers are needed to help understand the mechanism of disease onset, progression, prognosis and monitoring of the therapeutic response. Aim of this study was to identify the candidate circulating markers of chronic-phase chronic myeloid leukemia (CP-CML) manifestations, having potential to develop into predictive- or monitoring-biomarkers. A proteomic approach, two-dimensional gel electrophoresis in conjunction with mass spectrometry (2DE-MS), was employed for this purpose. Based on the spot intensity measurements, six proteins were found to be consistently dysregulated in CP-CML subjects compared to the healthy controls [false discovery rate (FDR) threshold ≤0.05]. These were identified as α-1-antichymotrypsin, α-1-antitrypsin, CD5 molecule-like, stress-induced phosphoprotein 1, vitamin D binding protein isoform 1 and transthyretin by MS analysis [PMF score ≥79; data accessible via ProteomeXchange with identifier PXD002757]. Quantitative ELISA, used for validation of candidate proteins both in the pre-treated and nilotinib-treated CP-CML cases, demonstrate that CD5 molecule-like, transthyretin and alpha-1-antitrypsin may serve as useful predictive markers and aid in monitoring the response of TKI-based therapy (ANOVA p < 0.0001). Two of the circulating marker proteins, identified in this study, had not previously been associated with chronic- or acute-phase myeloid leukemia. Exploration of their probable association with CP-CML, in a larger study cohort, may add to our understanding of the disease mechanism besides developing clinically useful biomarkers in future.

The recent release of the draft map of human ‘tissue proteome’^1^ has rekindled the interests of many research groups for parallel mapping and annotation of the human ‘fluid proteome’ primarily due to the existence of small but noticeable fractions of proteins that are exclusively present in the bodily fluids such as blood plasma/serum, urine, CSF, etc.^2^. These proteins may otherwise escape easily from detection, if only ‘tissue’ is employed as a ‘sole source’ for proteome investigation, in a diseased state. While the efforts for human plasma proteome profiling was initiated back in 2002 by the Human Proteome Organization (HUPO)^3^, the dynamic abundance of circulating proteins and intrinsic person-to-person inconsistencies in the expression patterns and/or release profiles under different physiological and patho-physiological conditions, made the plasma proteome mapping and the discovery of ‘universally-acceptable’ circulating biomarkers, a rather challenging task. The limitations therefore necessitate the building up of the individual, disease-specific proteome maps with enrollment of samples from diverse population groups to ensure a better identification of diagnostic-, predictive-, prognostic- and/or therapy-associated biomarkers.

Chronic myeloid leukemia (CML) is a myeloproliferative neoplasm, which results from reciprocal translocation between chromosome 9 and 22 t(9;22) (q34;q11) [Philadelphia chromosome] generating *BCR-ABL*, a tyrosine kinase encoding oncogene^4^. During the course of CML progression (chronic-, accelerated- and blast-crises phases), underlying gradual amplification of *BCR-ABL*-driven genomic instability and secondary modifications at genetic/epigenetic levels are believed to have major knock-on effect in altering and activating the expression of different mitogenic, anti-differentiating and anti-apoptotic modulators and mediators with resultant profound influence on the proteome profiles^4^,^5^,^6^. Analysis of these altered protein profiles in patients and their healthy counterparts are likely to assist in keeping track of the underlying concealed perturbations while expediting the search for novel diagnostic biomarkers and therapeutic targets of the disease.

This study is designed for comparative plasma proteome analysis of chronic phase-chronic myeloid leukemia (CP-CML) patients to meet two-fold objectives i.e., 1) identify novel, differentially expressed proteins in peripheral blood plasma having potential to develop into predictive- or therapy-associated biomarkers, and 2) make modest yet significant contribution in the international efforts for building up of a precise, accurately annotated universal plasma proteome map by providing protein data from the South Asian region, in particular Pakistan. Findings of this work present two novel, potential candidate biomarkers of myeloid leukemia.

## Results

### 2D proteome profiling and mass spectrometric analysis

**Figure 1:**
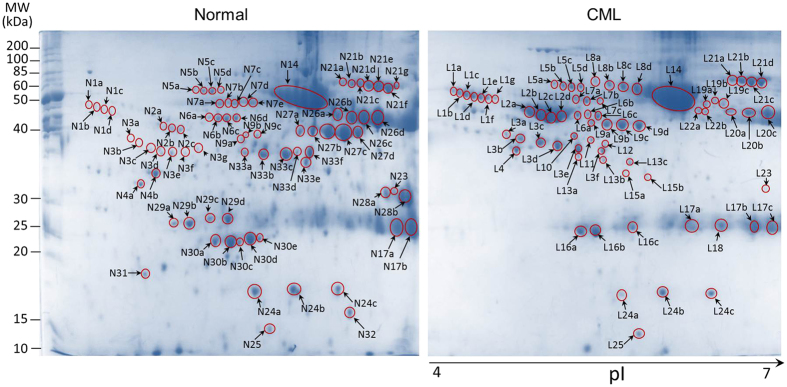
2D-gel images of plasma samples derived from normal and CP-CML subjects. Representative gel images of three independent experiments were merged to have a composite map in the pH range 4 to 7. Protein spots were visualized by staining with colloidal Coomassiee brilliant blue G250 and are numbered with N- and L-labels for normal and CP-CML samples, respectively. The protein spots showing at least one-fold difference were only considered for identification by MS analysis; those exhibiting minor or inconsistent changes were ignored and are therefore unlabeled.

Plasma proteins of the healthy and CP-CML subjects, enrolled in this study, were individually resolved by 2DE in three independent experiments, over a pH range 4 to 7 (Fig. 1, Tables 1 and 2). On average, 198 ± 76 spots in the healthy and 172 ± 83 spots in the CP-CML plasma appeared, when the 2D-gels were stained with colloidal-Coomassie. Altogether 68 ± 11 gel spots showed at least one-fold difference in intensities [as assessed by Dymension software (v 3.0.1.2)] and were therefore considered for MS analysis; those exhibiting minor or inconsistent changes were ignored. From the pooled control and the individual CP-CML samples, ~1300 gel spots were subjected to MALDI-TOF MS analysis, which led to the identification of 33 distinct proteins and/or their respective isoforms/subunits (Table 3). To address reliability issue of the peptide mass fingerprinting identification, PMF score for individual identifications was calculated using 79 as the cut-off value for positive hits^7^. The proteomics data was deposited to the ProteomeXchange Consortium^8^ via PRIDE partner repository (http://www.ebi.ac.uk/pride/) with the dataset identifier PXD002757.

**Figure 2:**
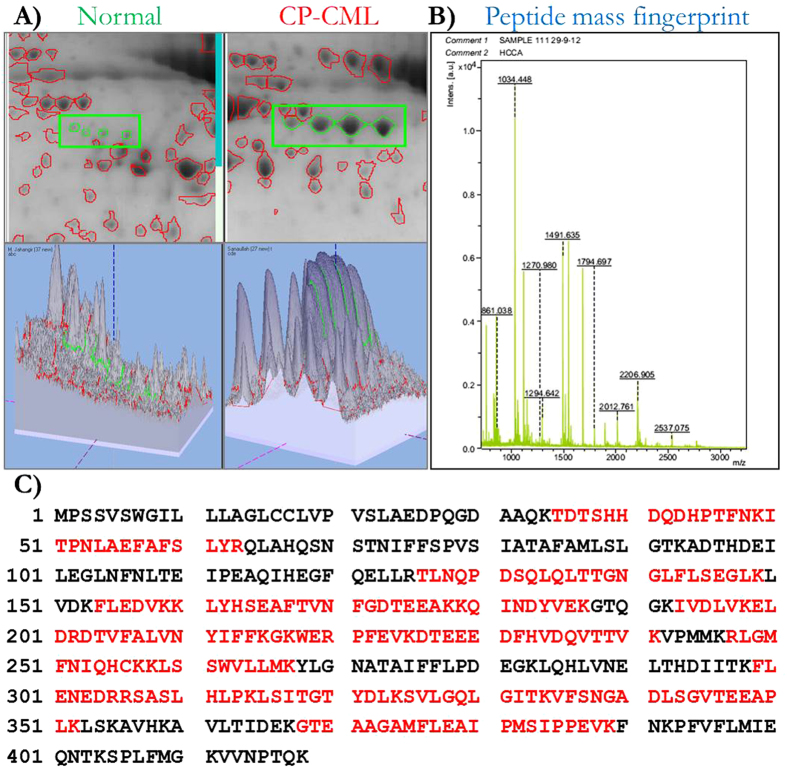
3D-simulation and mass spectrometry based identification of representative protein spots. (**A**) Upper panel shows the image of encircled 2-D gel spots in healthy and CP-CML subjects while the lower panel shows their corresponding 3D-images obtained using Image Master 2D-Platinum software; difference in spot intensities amongst the two study subjects are clearly visible. (**B**) Peptide mass fingerprint data of selected protein spot obtained following MALDI-TOF/TOF mass spectrometry analysis. (**C**) Identification of protein using online MASCOT program; matched peptide sequences of identified protein having sequence coverage of 55%, are shown in red bold.

When analyzed, majority of the identified proteins were represented by multiple spots in the CP-CML and the control groups (Fig. 1, Table 3). For instance, alpha-1-antitrypsin (AAT) and alpha-1-antichymotrypsin (AACT) are represented by 4 and 7 spots, respectively, in the CP-CML samples while the same proteins were represented by 3 and 4 spots in the healthy counterparts (Fig. 2). Slight to moderate pI or mass shifts between theoretical and experimentally-calculated values were also noticed. These observations seem to be the result of post-translational modifications such as phosphorylation, glycosylation and/or proteolytic cleavage that are likely to affect the electrophoretic mobility, stability, folding and interactions of the proteins and may be responsible for different protein isoforms. To substantiate that the discrepancy observed in the mass or pI is due to the glycosylation (a widely observed, structurally diverse event), the peptide mass fingerprinting data was subjected to the N-linked glycosylation analysis using NetNGlyc 1.0 (http://www.cbs.dtu.dk/services/NetNGlyc/) webserver. The glycosylation was predicted in many of the identified protein sequences namely AACT, AAT, VDBP, HP, etc., with very high scores (threshold ≥ 0.5) suggesting that mass and pI shifts in these proteins may be attributed to post-translational glycosylation (Supplementary data 1).

Prior to identifying the differentially-represented proteins we, therefore, summed up the intensities of the multiple spots of the same protein and applied the paired t-test followed by false discovery rate (FDR) determination as described previously^9^. The cut-off value for FDR (the probability of expected type 1 error in null hypothesis) was set as ≤0.05 to demonstrate that 95% findings are accurate. Only six proteins qualified the three-tier criteria that was set for screening of potential candidate biomarkers [p-value ≤ 0.05, FDR ≤ 0.05, PMF score ≥79; Table 3] and these were AAT, AACT, stress-induced phosphoprotein 1 (STIP1), CD5 molecule-like (CD5L), transthyretin (TTR) and vitamin-D binding protein (VDBP). Former four proteins were found at higher abundance while the later two showed decreased levels in colloidal Coomassie-stained gels of CP-CML in comparison with the control group. We selected all six differentially-represented proteins for further validation along with two statistically-insignificant/invariable proteins [haptoglobin (HP) and fibrinogen γ (FGG)], as control (Table 3).

### Immunological validation of candidate marker proteins

Validation of candidate proteins in the pre- and post-treatment CP-CML patients was performed using quantitative ELISA. Blood samples from 17 patients, out of the 32 initially enrolled subjects, who had undergone Tyrosine Kinase Inhibitor (TKI)-based therapy (nilotinib) for one year, were redrawn. Other patients (n = 15) could not become the part of this follow-up study either because of their demise or non-traceability.

**Figure 3:**
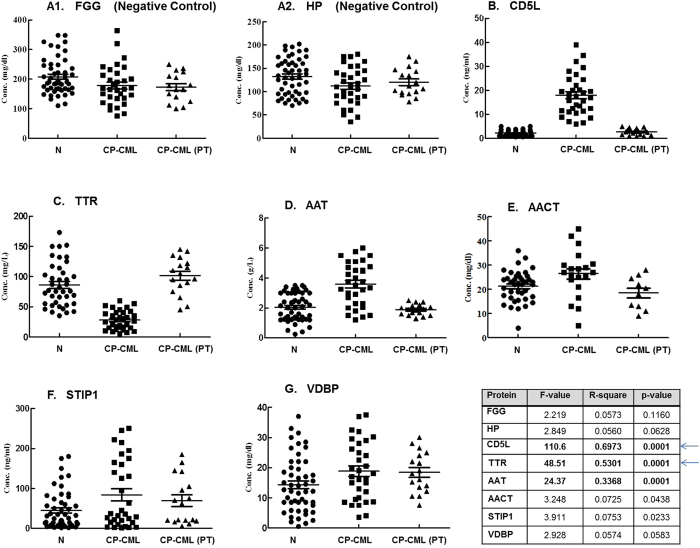
ELISA-based quantitative estimations of (**A1**) fibrinogen gamma (FGG), (**A2**) haptoglobin (HP), (**B**) CD5 molecule-like (CD5L), (**C**) transthyretin (TTR), (**D**) alpha-1-antitrypsin (AAT), (**E**) alpha-1-antichymotrypsin (AACT), (**F**) stress-induced phosphoprotein 1 (STIP1) and (**G**) vitamin-D binding protein precursor (VDBP) in plasma samples of normal (N) and chronic-phase CML (CP-CML) subjects. PT denotes CP-CML cases that have undergone TKI-based therapy (nilotinib treatment) for one year. One-way ANOVA p-value and F-value were calculated using SPSS program.

Except VDBP, all the candidate proteins showed differentiating expression patterns as manifestation of the disease (Fig. 3). More importantly, mean plasma concentration of CD5L in the pre-treated CP-CML subjects was 16.60 ± 7.99 ng/ml, which is nearly seven-times higher than the control group (2.29 ± 1.23 ng/ml). In the nilotinib-treated CP-CML (PT) cases, the normal levels of CD5L were, however, restored [(2.77 ± 1.37 ng/ml), Fig. 3B]. Likewise, prior to treatment, the patients group showed down-regulated expression of plasma TTR but they regained the normal levels following nilotinib therapy (Fig. 3C). The response of other candidate proteins viz. AAT, AACT and STIP1, in pre- and post-treatment CP-CML subjects was also not different from the above two markers; their ANOVA p-value was, however, higher than 0.0001 (Fig. 3D–F).

### *In-silico* characterization and pathway analysis

**Figure 4:**
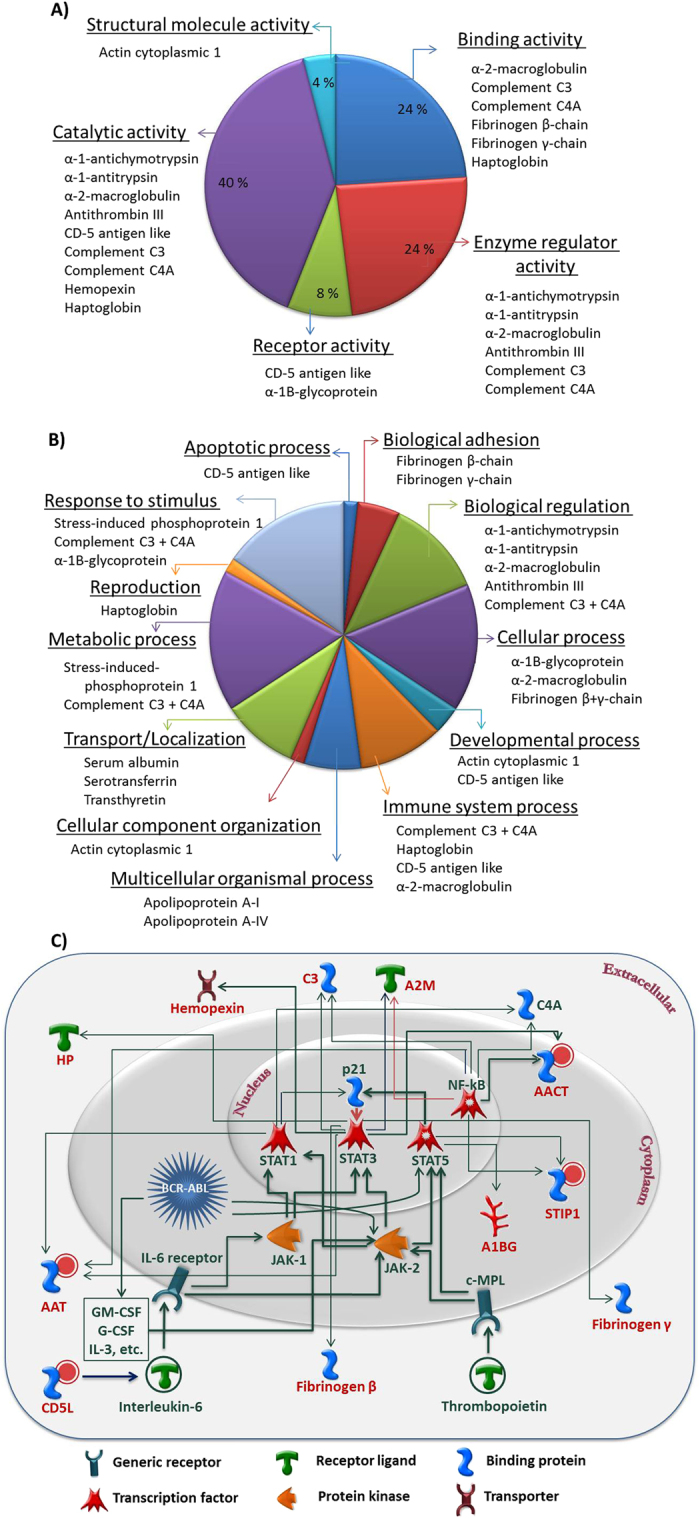
Classification of identified protein according to their (**A**) molecular functions and (**B**) biological processes. The assignments are based on Gene Ontology (GO) consortium (www.geneontology.org). (**C**) Network analysis of the MS identified differentially-abundant proteins in the dataset. The curated pathway is supported by at least one reference in the literature. Individual proteins are represented as nodes where shapes represent the fundamental class to which the proteins belong to. Small circle on-top of the protein symbols (red color) point towards an up-regulated response. Connecting lines between nodes define activation (green) and inhibition (red) while their thickness symbolizes the strength of interaction.

The molecular functions and biological processes, in which the MS identified proteins are involved in, according to the Gene Ontology database, were analyzed (Fig. 4A,B). As shown, majority of the proteins belong to the category of enzymes (9%), enzyme modulators (18%), transfer/carrier proteins (9%), immunity/defense proteins (15%), receptors (6%) and/or signaling molecules (15%). Interactive links between 11 such proteins could be traced using STRING and MetaCore^TM^ programs and are illustrated in the form of a curated pathway (Fig. 4C). This curated pathway was used as scaffolding to establish association of the elevated levels of plasma CD5L, AAT, AACT, STIP1 etc. in Philadelphia positive CP-CML cases.

Although it is difficult to speculate the exact correlation of each protein or node, nonetheless our results reinforce the earlier findings that the BCR-ABL constitutive tyrosine kinase activity exerts strong influence on the apoptotic- and immunity/defense-related biofunctions^10^,^11^. This oncoprotein activates many signaling cascades including the Janus kinase (JAK) signal transducers and activators of transcription (STAT) pathway, a pathway that is frequently triggered in both acute and chronic forms of myeloproliferative diseases. Besides activating the JAK-STAT, BCR-ABL induces the production of JAK2-activating cytokines viz. interleukin-3 (IL-3), IL-6, granulocyte macrophage colony stimulating factor (GM-CSF), G-CSF, etc. This cytokine enriched microenvironment is capable of activating the STAT3 and STAT5 signaling pathways via JAK-2, in a BCR-ABL independent fashion^10^,^11^,^12^. Thus, elevated levels of circulating STIP1, AACT, AAT and CD5L, in the present study, are likely to be the consequence of aberrant STAT signaling and constitutive activation of STAT3 and STAT5.

## Discussion

Myeloproliferative neoplasm CML is clinically diagnosed using a combination of complete blood cell count, molecular/cytogenetic testing and bone marrow aspiration and biopsy; blood-based protein biomarkers for screening- or monitoring the therapeutic response are, however, lacking. During the past decade, proteomic- and metabolomic approaches encompassing comparative analysis of proteins, peptides or small metabolites in healthy and diseased states has aided the discovery of several hundred candidate biomarkers of cancer diagnosis and/or prognosis and hence provided better insight into the disease mechanisms^13^,^14^,^15^. With an objective of identifying the robust, clinically-applicable, blood-based protein biomarkers of CML, we have compared the plasma proteome profiles of CP-CML subjects and their healthy counterparts using 2DE in conjunction with MALDI-TOF MS.

During the initial screening, eighteen proteins were found differentially-represented with FDR value ≤ 0.1 (90% confidence for accuracy) and amongst these six proteins displayed differential staining with better FDR value ≤ 0.05 (95% confidence for accuracy). These six proteins (Table 3, shown in bold) were selected for further validation, wherein except VDBP, all candidate biomarkers showed potential to discriminate the healthy control group from the patients and the pre-treatment cases from the post-treatment CP-CML group. AAT, TTR and CD5L proteins with ANOVA p-value ≤ 0.0001 appears to be of particular interest as they were better able to predict the patients’ clinical behavior and therapeutic response.

AAT, a 54 kDa glycoprotein is a serine protease inhibitor, which earlier has been described to be associated with tumor progression and metastasis in a wide spectrum of cancers including CML^16^,^17^. There is, however, no direct evidence in the literature showing the association of myeloid leukemia (either CML or AML) with differential abundance of TTR and/or CD5L. Thus not AAT but the other two proteins appear to be novel. Amongst these, TTR is an extracellular protein which is synthesized in the liver and is involved in the transport of thyroxin from blood to brain besides acting as a carrier of retinol^18^. In comparison with Chinese healthy subjects wherein plasma TTR levels have been reported as 129 ± 15.6 μg/ml^19^, our healthy control group showed significantly lower levels both in males (108 ± 31.99 μg/ml) and females (63.48 ± 24.29 μg/ml). Pronounced gender-associated differences in circulating TTR concentrations are also obvious. This is not surprising as many proteins including haemoglobin have shown gender-related differences in clinical settings. Much lower TTR levels in our control group, however, are somewhat interesting because Liu *et al*.^19^ proposed an optimal cut-off value of 115- and 88.5 μg/ml, respectively to discriminate the healthy subjects from those suffering from benign lung diseases and the lung cancer. Similar cut-off, if applied on our population, where even healthy females have circulating TTR lower than the threshold value set for the lung cancer diagnosis, is likely to result in large number of false-positives. This calls for the need of plasma proteome profiling from diverse population groups of variable ethnicity to ensure discovery of better and universally acceptable biomarkers.

Another interesting biomarker identified in this study is CD5L, a 347 amino acid long soluble, secreted protein. It is a member of SRCR superfamily, which is characterized by the presence of scavenger receptor cysteine rich (SRCR) domains with critical roles in lipid homeostasis, inflammation and immune responses^20^. In the validation study, the plasma concentration of CD5L was found significantly elevated in the CP-CML group, which dropped to the normal levels following TKI-based therapy (ANOVA p-value ≤ 0.0001 and F-value = 110.6), suggesting the effectiveness of candidate marker in monitoring the therapeutic-response as well.

In the Human Protein Atlas (http://www.proteinatlas.org), most of the cancer types such as breast, colorectal, head and neck, cervical, lung, liver, prostate, ovarian cancer, etc., were found negative for the presence of CD5L making it a specific biomarker of leukemia. However, it is of relevance that CD5L is a secretary protein and the tissue analysis may not accurately portray its expression profile. More so, significantly high levels of circulating CD5L has been reported in the patients suffering from pulmonary tuberculosis^21^, liver cirrhosis with HCV infection^22^,^23^ and hepatocellular carcinoma with non-alcoholic fatty liver disease^24^. We have noted that the CML patients are generally immune-compromised and majority of them suffer from hepatomegaly and/or splenomegaly [Table 2]. The question whether elevated levels of circulating CD5L in CP-CML reflects a coordinated response of infection and inflammation or relates to myeloid leukemia as a function of anti-apoptotic factor, suggests large-scale trials with enrolments of lymphoid- and myeloid-leukemia (ALL, CLL, AML, CML) patients from diverse population groups.

Taken together, in complex diseases such as cancers/leukemia, a single protein or peptide is unlikely to serve as disease biomarker in all population groups. AAT, TTR and CD5L, however, have shown potential to serve as predictive- or therapy-associated CP-CML biomarkers. Further investigation of their specific role and the cross-talk amongst the repertoires of immune- and apoptotic-effectors is likely to provide new clues about the cellular biology of myeloid leukemia.

## Methods

### Study population

The study population was comprised of 82 subjects in total that included healthy controls (n = 50, Table 1), BCR-ABL positive CP-CML subjects (n = 32, Table 2) and post-treatment CP-CML cases (n = 17; received nilotinib therapy for a period of one year). Informed consent was obtained from all subjects, prior to their enrolment in the research project. The study design was duly approved by the Ethical Review Committee of the School of Biological Sciences, University of the Punjab, Lahore, Pakistan [Ref. No. 873/12] and was in accordance with the principals of the Declaration of Helsinki for research involving human beings. The peripheral blood samples (3cc) from healthy donors and the CP-CML patients were collected in EDTA-coated tubes, centrifuged at 2,000 × g for 10 minutes to separate plasma and then stored at −80 °C, in 250 μl aliquots, until their use for analysis. The samples were processed within 30 minutes after collection.

### Fractionation of proteins by two-dimensional gel electrophoresis

Total protein contents in the collected plasma samples were estimated by Bradford assay^25^ using bovine serum albumin (BSA) as standard. Applying 2D-gel electrophoresis, protein fractionation was performed according to the procedure described previously with minor modifications^26^. Briefly, the plasma sample was diluted with rehydration solution [7 M urea, 2 M thiourea, 2% CHAPS, 65 mM DTT and 0.25% Servalyte] to a concentration of 1 μg/μl and applied onto Servalyte 18 cm long, linear immobilized pH gradient (pH 4–7) strip (Serva Electrophoresis, Heidelberg, Germany). The dried strip was subjected to passive rehydration overnight at 20 °C and then focused on IEF flatbed (IEF-SYS, SciePlas, UK) for a total of 60kVhr.

Following first dimension IEF, strips were successively equilibrated with equilibration buffer-I [6 M urea, 2% SDS, 30% glycerol and 1% DTT in 1.5 mM Tris-Cl (pH 8.8)] and buffer-II [6 M urea, 2% SDS, 30% glycerol, 5% iodoacetamide in 1.5 mM Tris-Cl (pH 8.8)], each for 15 minutes. Equilibrated strips were aligned on 12% SDS-gel and electrophoresed at 80 V initially for 1 hour and then at 160 V until the bromophenol blue tracking dye reached the bottom of the gel. After electrophoresis, the gel was placed in fixative solution (30% ethanol, 10% acetic acid) overnight, stained with Coomassie colloidal blue dye and then destained with deionized water to a clear background. 2D gel images were scanned using Syngene gel documentation system and the individual protein spots were analyzed for pI and molecular weight, followed by their quantification and matching using the Dymension v.3.0.1.2 (Syngene, UK) software program.

### In-gel digestion and mass spectrometric analysis

After matching the proteins of healthy and CP-CML subjects, individual gel spots were excised under sterile conditions, washed twice with deionized water, and then destained completely by incubation with 100 μl of 0.2 M ammonium biocarbonate (AB) and 50% acetonitrile solution (1:1) at 37 °C for 30 min. Proteins in gel spots were thereafter reduced and alkylated by successive incubations with 100 μl 20 mM tris (2-carboxyethyl) phosphine containing 25 mM AB and 40 mM iodoacetamide containing 25 mM AB, each at 37 °C for 30 min. in the dark. The gel pieces were washed with 100 μl of 5 mM β-mercaptoethanol containing 25 mM AB for 15 min. at 37 °C and dried completely in a speed vac. For tryptic digestion, the gel slices were rehydrated with 20 μl of 0.02 mg/ml sequencing grade trypsin (Promega, V511A) and left for overnight digestion at 37 °C. Resulting peptides were extracted from the gel by centrifugation, washed with 40 mM AB/acetic acid (incubation 37 °C for 30 min.) and spotted on target plate for mass spectrometric analysis.

For MALDI analysis, 1 μl of the digested peptides was mixed with equal volume of freshly prepared saturated solution of α-cyano-4-hydroxycinnamic acid prepared in 0.1% triflouroacetic acid/acetonitrile. 1 μl of this mixture was then spotted on to the target plate, air dried until solvent evaporation, and then analyzed using MALDI-TOF-TOF MS (Ultraflex III, Bruker Daltonics, Germany). A 337 nm nitrogen laser and a 2 GHz digitizer were used at a laser frequency of 100 Hz and an intensity of 60–70%. Spectra were obtained in linear positive ion mode with accelerating voltage of 25 kV and lens potential of 6 kV. Delayed extraction was performed at 100 ns, the detector gain was set to 7.5 and the sample rate to 0.5 GS/s. Spectra were obtained in the mass to charge (*m/z*) range of 1000–5000.

### Functional and pathway analysis of proteins using bioinformatics tools

Peptide mass spectra obtained from MS analysis were searched against the SWISS-PROT and NCBInr databases using the MASCOT Wizard 1.1.2. from Matrix Science (www.matrixscience.com) and further confirmed by MS-fit program from Protein Prospector (www.prospector.ucsf.edu). The proteins identified with PMF score 79 or higher were considered as acceptable (27). Search parameters included *Homo sapiens* as species with methionine oxidation and carboxymethylation of cysteine residues as the variable and the fixed modifications, respectively, and an allowable peptide mass tolerance of 50–100 ppm. Enzymatic digestion of proteins was performed with trypsin (V115, Sigma Aldrich, USA). The identified proteins were categorized according to their GO (www.geneontology.com) annotations based on molecular functions. Network construction analyses and canonical pathways were generated through the use of MetaCore^TM^ (Functional Genomics Centre, University of Zurich, Switzerland) and protein functional analysis software, STRING v.9.1 (string.db.org).

### Statistical analysis and validation of biomarker candidates

The statistical analyses were performed using SPSS ver.20 and/or GraphPad Prism 6.01 software programs. The spot intensity differences obtained from the 2D-gel images of at least 3 different sets of independent plasma samples were analyzed by non-parametric Mann-Whitney *U* test. Proteins with high fold-changes (≥2.5) were considered for validation studies by enzyme linked immunosorbant assays (ELISA). To minimize sample handling bias or pipetting errors, pre-coated ELISA plates against human AAT (also called SERPIN A1), AACT (also known as SERPIN A3), TTR (also called as pre-albumin), CD5L, STIP1, VDBP, HP and FGG were obtained from GenWay Biotech Inc., CA, USA and Biomatik Corporation, USA, and used in the validation of candidate biomarkers. All the assays were performed in triplicates according to the recommended instructions of the supplier. One-way analysis of variance (ANOVA) with an unpaired Student's t-test (applied to check the significance of differences amongst the group mean values) was applied to compare the antibody titer between test and the control groups. Associations with a *p*-value ≤ 0.05 were considered as statistically significant, while those with *p*-value ≤ 0.0001, as clinically important.

## Additional Information

**How to cite this article**: Fatima, I. *et al*. CD5 molecule-like and transthyretin as putative biomarkers of chronic myeloid leukemia - an insight from the proteomic analysis of human plasma. *Sci. Rep.* **7**, 40943; doi: 10.1038/srep40943 (2017).

**Publisher's note:** Springer Nature remains neutral with regard to jurisdictional claims in published maps and institutional affiliations.

## Supplementary Material

Supplementary Dataset 1

## Figures and Tables

**Table 1:** Hematological profile of healthy subjects (n = 50).

	Hb (g/dl)	WBC (x10^3^/μl)	RBC (x10^6^/μl)	PLT (x10^3^/μl)	Lymphocytes (%)	Monocytes (%)	Eosinophils (%)	Neutrophils (%)	Basophils (%)
Minimum	11.50	4.96	3.80	175.00	18.30	4.20	1.50	30.50	0.30
Maximum	14.80	8.57	5.07	386.00	50.60	9.90	8.00	73.90	1.30
Mean	13.26	6.44	4.47	260.33	34.15	6.59	3.31	53.86	0.79
Std. Deviation	1.06	1.30	0.38	63.61	8.60	1.52	1.56	10.68	0.34

**Table 2:** Clinical manifestations of the patients enrolled in the study.

CP-CML Patients	n = 32
Age at diagnosis	
Median	37 (17–45 years)
≤37	18
≥37	14
Therapy received (chemo/radio)	None
Family History	None
Lymphadenopathy	
Cervical	5
Inguinal	11
Axillary	4
No	12
Hepatomegaly (Y/N)	
Yes	15
No	17
Splenomegaly (Y/N)	32
Yes	
Hematological profile	
Hemoglobin (*Ref. 11.5–17.5 g/dl*)	1.5–10.3 g/dl
WBC count (*Ref. 4–11 *×* 10*^*3*^*μl*)	48–525 × 10^3^ μl
Platelet count (*Ref. 150–400 × 10*^*3*^*μl*)	53–289 × 10^3^ μl
Lymphocytes (*Ref. 20–45%*)	1–20%
Monocytes (*Ref. 2–10%*)	1–5%
Eosinophils (*Ref. 0–6%*)	1–7%
Neutrophils (*Ref. 40–75%*)	29–52%
Basophils (*Ref. 0–1.5%*)	0–1%

**Table 3:** List of proteins identified in the plasma samples of controls and the CP-CML subjects by MALDI-TOF MS.

Sr. No	Protein Name	Accession Number	Spot No.	Expression	*p-*value	FDR*	pI		Approx. MW (kDa)		Mascot Score	Sequence Coverage (%)	Mass values searchd	Mass values matched	PMF Score**
							Observed	In Database	Observed	In Database					
**1**	**α-1-antichymotrypsin**	**gi|1340142**	**L1a-gN1a-d**	**2.96 ± 1.060.84 ± 0.50**	**0.021**	**0.05**	**4.53–4.84**	**5.34**	**71–76**	**47.79**	**64**	**25**	**68**	**9**	**205.15**
**2**	**α-1-antitrypsin**	**gi|177831**	**L2a-dN2a-c**	**10.04 ± 4.1403.20 ± 2.21**	**0.004**	**0.04**	**5.04–5.23**	**5.43**	**62–66**	**46.84**	**210**	**55**	**93**	**28**	**255.86**
3	Haptoglobin	gi|3337390	L3a-fN3a-g	7.58 ± 3.515.91 ± 3.87	0.482	0.48	4.88–5.54	6.14	44–52	38.72	110	35	71	14	163.25
4	Chain C, Human complement component C3c	gi|78101271	L4N4a,b	0.93 ± 0.310.84 ± 0.62	0.102	0.15	4.96	4.79	45	40.20	107	40	63	14	138.28
5	α-1-β glycoprotein	gi|119592981	L5a-dN5a-d	0.93 ± 0.250.61 ± 0.22	0.165	0.19	5.21–5.41	5.58	79–81	54.81	70	22	67	10	126.96
**6**	**Vitamin D-binding protein isoform 1 precursor**	**gi|32483410**	**L6a-cN6a-d**	**0.78 ± 0.161.21 ± 0.27**	**0.021**	**0.05**	**5.38–5.54**	**5.32**	**61–62**	**54.48**	**113**	**28**	**70**	**15**	**146.65**
7	Antithrombin III variant	gi|576554	L7a-cN7a-e	1.98 ± 1.223.53 ± 2.77	0.177	0.19	5.30–5.45	6.11	70–71	53.11	69	25	39	11	121.47
8	Hemopexin precursor	gi|11321561	L8a-dN8	2.65 ± 1.211.20 ± 0.94	0.049	0.08	5.42–5.67	6.55	80–85	52.39	106	34	49	16	140.5
9	Fibrinogen γ	gi|223170	L9a-dN9	4.85 ± 0.792.71 ± 1.70	0.025	0.09	5.48–5.79	5.54	54–56	46.82	134	31	45	15	147.81
10	Apolipoprotein A-IV precursor, partial	gi|178779	L10N10	0.16 ± 0.170.04 ± 0.04	0.102	0.24	5.32	5.22	48	43.36	162	53	82	22	119.78
11	Actin, β, partial	gi|14250401	L11N11	0.16 ± 0.070.07 ± 0.10	0.205	0.36	5.48	5.56	47	41.32	100	32	74	11	128.12
**12**	**CD5 molecule-like**	**gi|5174411**	**L12N12**	**0.21 ± 0.140.02 ± 0.03**	**0.003**	**0.01**	**5.57**	**5.28**	**48**	**39.60**	**61**	**35**	**39**	**11**	**142.06**
13	Haptoglobin isoform 2 preproprotein	gi|186910296	L13a-cN13	0.94 ± 0.600.33 ± 0.22	0.097	0.32	5.38–5.72	6.13	43–46	38.94	73	20	26	8	108.48
14	Serum albumin	gi|332356380	L14N14	30.58 ± 3.8629.07 ± 8.45	0.273	0.36	5.99	5.73	70	68.48	186	45	85	30	186.11
15	Apolipoprotein E	gi|4557325	L15a, bN15	0.17 ± 0.150.15 ± 0.08	0.430	0.43	5.72–5.86	5.65	33–34	36.25	89	36	41	11	139.86
16	Chain A, crystal structure of lipid-free human apolipoprotein A-I	gi|90108664	L16a-cN16	2.92 ± 1.102.16 ± 1.86	0.220	0.36	5.41–5.75	5.27	19–21	28.06	174	69	69	20	188.26
17	Immunoglobulin light chain	gi|149673887	L17a-cN17a,b	9.37 ± 3.207.84 ± 1.23	0.243	0.36	6.01–6.73	6.97	22–25	23.67	80	36	47	6	79.32
18	Ras-related protein Rab-37 isoform 3	gi|28376635	L18N18	1.79 ± 1.751.81 ± 0.36	0.252	0.36	6.37	5.97	23	24.27	67	49	45	12	108.49
19	α-2-macroglobulin, partial	gi|177872	L19a-cN19	0.29 ± 0.170.66 ± 0.67	0.080	0.32	6.24–6.37	5.47	66–68	71.32	84	23	52	13	161.40
20	Chain B, crystal structure of human fibrinogen	gi|237823915	L20a-cN20a-f	3.74 ± 0.882.61 ± 2.37	0.319	0.36	6.45–6.71	7.14	61–66	52.91	177	55	84	26	290.0
21	Serotransferrin	gi|313104271	L21a-d	5.55 ± 1.836.50 ± 1.65	0.364	0.364	6.48–6.71	6.81	84–87	79.29	127	29	59	21	218.49
**22**	**Stress-induced-phosphoprotein 1**	**gi|297688341**	**L22a,b**	**0.27 ± 0.36**	**0.009**	**0.04**	**6.21–6.27**	**6.40**	**66–67**	**63.23**	**57**	**30**	**48**	**16**	**132.27**
23	Chain C, complement C4 in complex with Masp-2	gi|401871713	L23N23	0.35 ± 0.190.53 ± 0.16	0.055	0.09	6.68	6.37	34	33.74	82	35	34	9	128.31
24	Hap-2α/hp2-α	gi|296653	L24a-cN24a-c	2.75 ± 2.134.79 ± 2.08	0.042	0.09	5.67–6.25	6.25	15	42.13	90	29	100	12	94.19
**25**	**Transthyretin**	**gi|114319005**	**L25N25**	**0.25 ± 0.270.83 ± 0.32**	**0.004**	**0.02**	**5.76**	**5.52**	**12**	**15.06**	**111**	**61**	**20**	**7**	**94.19**
26	PRO2619	gi|11493459	N26a-d	4.15 ± 6.17	0.087	0.09	6.25–6.44	5.96	60	58.51	93	23	58	12	114.16
27	ALB protein	gi|27692693	N27a-f	3.82 ± 5.59	0.065	0.09	5.91–6.27	5.97	48–50	48.64	70	26	69	10	107.15
28	Immunoglobulin G1 Fc fragment	gi|5031410	N28a,b	1.72 ± 2.52	0.085	0.09	6.53–6.69	6.95	31	25.40	72	43	70	9	143.82
29	Chain C, crystal structure of recombinant human fibrinogen fragment D	gi|24987625	N29a-d	0.66 ± 1.03	0.042	0.09	5.09–6.08	5.86	26–30	35.49	79	30	66	10	105.85
30	Albumin, isoform CRA_j	gi|119626073	N30a-e	1.96 ± 2.88	0.066	0.09	5.4–5.59	6.40	24	26.91	141	38	41	14	114.01
31	Ras-related protein Rab-3D	gi|4759000	N31	0.73 ± 0.11	0.062	0.09	4.37	4.76	17–20	24.48	57	52	62	10	88.83
32	HPX protein	gi|13529281	N32	0.27 ± 0.39	0.076	0.09	6.4	6.45	12	29.06	66	35	34	12	115.47
33	Fibrinogen β-chain isoform CRA_e	gi|119625339	N33a-e	3.68 ± 2.24	0.071	0.09	5.47–5.87	6.95	40	40.16	170	59	78	24	244.39

Six proteins that qualified the three-tier criteria i.e., p-value < 0.05, FDR ≤ 0.05 and PMF score >79, are shown in bold.

^*^FDR determination (the probability of expected type 1 error in null hypothesis) was performed according to the method of Diz *et al*.^9^. Value ≤ 0.05 indicates that 95% findings are accurate/true.

^**^PMF score, for each identification, was calculated as described by Stead *et al*.^7^ using 79 as the cut-off value for positive hits.
