# Supplementary material for: CD5 molecule-like and transthyretin as putative biomarkers of chronic myeloid leukemia - an insight from the proteomic analysis of human plasma
Source: Sci Rep. 2017 Jan 24;7:40943. doi: 10.1038/srep40943 (PMC5259771; doi:10.1038/srep40943)
Supplement: Supplementary Dataset 1 [file srep40943-s1.doc]

## Title

## CD5 molecule-like and transthyretin as putative biomarkers of chronic myeloid leukemia - an insight from the proteomic analysis of human plasma

## Authors

## Iram Fatima^1^, Saima Sadaf^2*^, Syed Ghulam Musharraf^3^, Naghma Hashmi^3^, Muhammad Waheed Akhtar^1, 2*^

## Authors’ Affiliation

^1^School of Biological Sciences, University of the Punjab, Lahore-54590, Pakistan

^2^Institute of Biochemistry and Biotechnology, University of the Punjab, Lahore-54590, Pakistan

^3^Panjwani Center for Molecular Medicine and Drug Research, International Center for Chemical and Biological Sciences, University of Karachi, Karachi-75270, Pakistan

## ^*^ Corresponding authors:

Email: [sasadaf@hotmail.com](mailto:sasadaf@hotmail.com); [saima.ibb@pu.edu.pk](mailto:saima.ibb@pu.edu.pk)

mwasbs@pu.edu.pk

**Table: Prediction of N-glycosylation in plasma proteins identified in CP-CML subjects.**

| **Sequence name** | **Position** | **Potential*** | **Jury agreement** | **N-Glyc result**** |
| --- | --- | --- | --- | --- |
| AACT | 33 NLTQ | 0.7096 | (8/9) | + |
|  | 93 NTTL | 0.8390 | (9/9) | +++ |
|  | 106 NLTE | 0.6910 | (9/9) | ++ |
|  | 127 NQSS | 0.6887 | (9/9) | ++ |
|  | 186 NGTR | 0.3967 | (7/9) | - |
|  | 271 NASA | 0.4458 | (6/9) | - |
| AAT | 70 NSTN | 0.7166 | (9/9) | ++ |
|  | 107 NLTE | 0.7610 | (9/9) | +++ |
|  | 271 NATA | 0.6390 | (9/9) | ++ |
|  | 414 NPTQ | 0.5552 | (7/9) | + |
| HP | 125 NLTT | 0.6580 | (8/9) | + |
|  | 148 NHSE | 0.6221 | (9/9) | ++ |
|  | 152 NATA | 0.5766 | (6/9) | + |
|  | 182 NYSQ | 0.7227 | (9/9) | ++ |
| VDBP | 288 NLST | 0.5713 | (7/9) | + |
| FGG | 52 NKTS | 0.7553 | (9/9) | +++ |

*Threshold was set as 0.5. Plus (+) sign indicates the higher potential of glycosylation.

**N-linked glycosylation analysis was performed using NetNGlyc 1.0 webserver (<http://www.cbs.dtu.dk/services/NetNGlyc/>).
